# CRISPR-Cas editing technologies for viral-mediated gene therapies of human diseases: Mechanisms, progress, and challenges

**DOI:** 10.1016/j.omtn.2025.102786

**Published:** 2025-11-27

**Authors:** Boris Kantor, Leanne Duke, Pradeep G. Bhide

**Affiliations:** 1 Viral Vector and Gene Editing Cores, Florida Institute for Pediatric Rare Diseases, College of Medicine, Florida State University, Tallahassee, FL 32306, USA; 2 Florida Institute for Pediatric Rare Diseases, College of Medicine, Florida State University, Tallahassee, FL 32306, USA; 3 Department of Biomedical Sciences, College of Medicine, Florida State University, Tallahassee, FL 32306, USA; 4 Department of Neurobiology, School of Medicine, Duke University, Durham, NC 27707, USA

**Keywords:** MT: Delivery Strategies, adeno-associated vectors, AAV, lentiviral vectors, clustered regularly-interspaced short palindromic repeats, CRISPR-associated protein 9, CRISPR-Cas9, epigenome-based editing, base editing, prime editing, all-in-one delivery system, gene therapy, gene delivery, clinical trials

## Abstract

The gene therapy landscape has evolved substantially in recent years, beginning with the approval of the first adeno-associated virus-based gene therapy, Luxterna, in 2017. Since then, the US FDA has approved nearly 30 new viral gene therapy programs, with notable examples including Zolgensma, Spinraza, Hemgenix, Zynteglo, Lyfgenia, Kymriah, Skysona, and Tecelra. Remarkably, all these products rely on delivery via adeno-associated vectors (AAVs) and lentiviral vectors (LVs). Improvements in viral-mediated gene transfer efficiency and clinical-scale manufacturing, together with immense commercial interest, have greatly propelled the clinical adoption of gene therapy products. In recent years, clustered regularly interspaced short palindromic repeats (CRISPR) and its related Cas proteins (CRISPR-Cas) have made significant advances in gene therapy, offering next-generation approaches for curative gene editing to treat genetic diseases and disorders. In this review, we examine the range of these therapeutics and their viral carriers, focusing primarily on LVs and AAVs. We provide a snapshot of the current status of the field and highlight some of the current challenges in the clinical application of gene therapy, with particular emphasis on viral CRISPR-Cas-based technologies and their future potential.

## Graphical abstract

**Figure:**
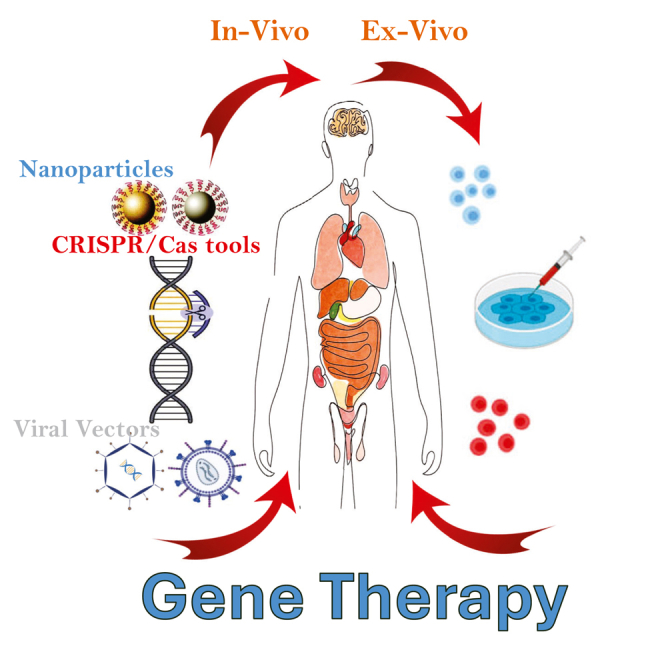

## Teaser

In this review, Kantor and colleagues highlight some of the current challenges faced in the preclinical and clinical uses of gene therapy, with particular emphasis on viral-CRISPR-Cas-based technologies and their future potential. They delve into the assortment of technologies and viral carriers, mostly focusing on lentiviral and adeno-associated vectors.

## Introduction

The advance of gene therapy programs is remarkable, with the last decade bearing witness to the approval of 29 gene therapy products (US Food and Drug Administration [FDA], “Approved Cellular and Gene Therapy Products | FDA”). The success of gene therapy for monogenic diseases is widely credited to adeno-associated vectors (AAVs), which have emerged as a cornerstone of the clinical landscape due to their effectiveness in therapeutic gene-to-cell transfer.^1^^,^^2^ The vast potential of AAV-based systems has been shown through their use in over four hundred clinical trials developed by more than two hundred biotech entities to treat over sixty-five diseases to date, along with the approval of seven AAV-based gene therapy programs, thereby positioning this vector platform at the forefront of the delivery field.^1^^,^^2^^,^^3^

Notably, the approval of Casgevy marks a remarkable milestone in the field of clinical therapy. This product, approved in 2023, became the first clustered regularly interspaced short palindromic repeats (CRISPR)-Cas9-based gene therapy drug for the treatment of patients with sickle cell disease (SCD), including those with recurrent vaso-occlusive crises (VOCs) and transfusion-dependent β-thalassemia (TDT).^4^ Remarkably, in 2024, the FDA approved nine new gene therapy drugs. These developments include chimeric antigen receptor (CAR) T-cell therapies as well as stem-cell and gene therapies. Among these treatments, three are based on viral-mediated gene delivery systems, including AAV and lentiviral vector (LV). Specifically, obecabtagene autoleucel (tradename Aucatzyl) was granted regenerative medicine advanced therapy designation and orphan drug designation following FDA approval of the drug in November 2024. This gene therapy product, developed by Autolus Inc., is an LV-based, CD19-directed, genetically modified autologous T cell immunotherapy designated for the treatment of adults with relapsed or refractory B cell precursor acute lymphoblastic leukemia (ALL) and systemic lupus erythematosus.^5^^,^^6^ Earlier that year, the FDA approved another LV-based gene therapy product, atidarsagene (Orchard Therapeutics’ Lenmeldy), indicated for the treatment of children with presymptomatic late infantile, presymptomatic early juvenile, or early symptomatic early juvenile metachromatic leukodystrophy (MLD).^7^ Finally, ladocagene exuparvovectneq (developed by PTC Therapeutics; commercial name, Kebelidi) represents the first-ever clinical drug approved for gene therapy directly administered to the brain. This AAV2.2 gene therapy product is designated for the treatment of adult and pediatric patients with aromatic L-amino acid decarboxylase (AADC) deficiency (clinical trial NCT04903288).

The main focus of this article is to critically review the prospects of two platforms used for the delivery of gene therapy and gene editing systems: AAVs and lentiviruses, concentrating on their current state and discussing emerging challenges related to their use for treating human diseases. However, it is worth noting that although long-term efficacy has been attained using these viral platforms in many clinical trials, concerns regarding immunogenicity, insertional mutagenesis, and oncogenicity associated with viral-mediated delivery still remain. Notably, with advances in material sciences and biomedical engineering, nonviral gene vectors play an ever-growing and important role in clinical gene therapy applications. Furthermore, due to the limitations of viral-based CRISPR-Cas delivery, nonviral methods are on the rise, particularly in clinical oncology (reviewed by Neshat et al. and Schock et al.^8^^,^^9^) and blood disorders (reviewed by Rana et al. and Zhou et al.^10^^,^^11^). Nonviral delivery approaches can generally be classified into physical or chemical carrier-mediated methods, which include cationic liposomes, lipid nanoparticles (LNPs), and polymers, and electroporation, gene gun, and nanomagnetic methods, respectively.^12^ The electroporation method can be further combined with synthetic ribonucleoprotein (RNP) formulations. RNPs deliver CRISPR components directly to a cell of interest rather than necessitating the transcription and translation steps required for viral-mediated counterparts (reviewed by Xu et al.^13^). Thus, nonviral methods offer superior control over how long the active components persist in cells and therefore may reduce undesirable off-target effects and toxicity. Although the efficiency of nonviral delivery methods used to date is still lower than that of their viral counterparts, their cost-effectiveness, manufacturing readiness, and most importantly, safer immunological profile, as well as the ability to package large genetic cargoes, will likely make them the next generation of delivery vehicles for a broad range of gene therapy applications. Further descriptions and complexities of nonviral delivery methods are beyond the scope of this discussion but have been excellently reviewed elsewhere.^14^^,^^15^

## Viral vectors for gene therapy

### Principles of viral-mediated gene transfer

Viral-mediated gene transfer represents an attractive technology for the delivery of therapeutic cargoes to the cell and tissue of interest. Importantly, it typically requires only a one-time, single-dose administration, whether for the replacement of a gene or genes for a hereditary disease or disorder or the delivery of genome-modifying therapy (GMT) for treatment. This is very appealing compared to some other treatments that require multiple doses. Thus, extensive effort has been dedicated to the development of improved and optimized viral vectors. Different vector systems are, of course, tailored to their specific applications and uses, but all viral-mediated delivery systems generally share several key features. Several important points must be checked before a system can become an efficient delivery vehicle. In fact, the ideal viral vector will integrate high packaging capacity, efficient transduction rates paired with long-term stable gene expression, and high efficiency in mediating gene-to-cell transfer in both dividing and nondividing cells and tissues; while lacking undesired insertional activity, maintaining low immunogenicity, and being highly suitable for inexpensive and scalable manufacturing.

### LVs: Basic biology and engineering

Historically, retroviruses, followed by LVs, have been the most commonly used type of gene delivery system for clinical gene therapy. Unlike simple g-retroviruses, lentiviruses evolved a mechanism that efficiently exploits a host-mediated pathway supporting nuclear translocation and import through the intact nuclear membrane.^16^ Subsequently, LVs evolved into highly efficient delivery vehicles, demonstrating high transduction efficiency in both nondividing and terminally differentiated cells, e.g., postmitotic neurons (reviewed by Byrne et al.^2^). The lentiviral genome occupies ∼10.8 kb of positive-sense single-stranded (ss) RNA, of which two identical copies are co-packaged inside a lipid-supplemented viral capsid that is ∼130 nm in diameter.

**Figure 1:**
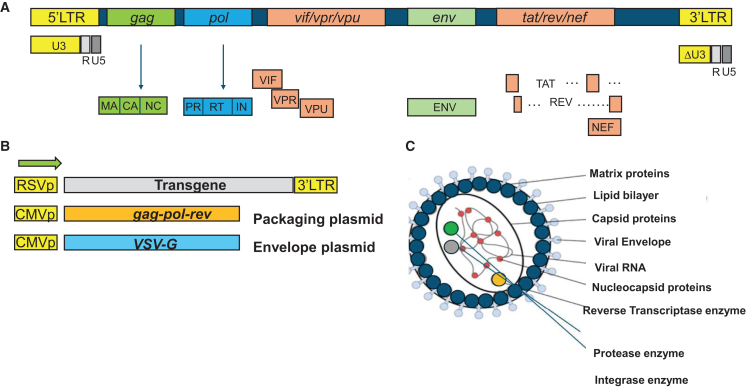
Overview of retroviral and lentiviral vectors (A) Simplified schematic of the wild-type human immunodeficiency virus type 1 (HIV-1) genome. (B) Lentiviral vector-split-genome system. The transgenic, envelope, and packaging plasmids are highlighted. (C) Structure of lentiviral vector particles. The viral genome, capsid, and envelope structures are schematically illustrated.

The recombinant lentiviral (rLV) systems have been engineered by removing all pathogenic or infectious genes from the wild-type HIV-1 virus,^17^ as diagrammed in Figures 1A and 1B. As such, the genome of the recombinant vector encodes only the essential structural and enzymatic genes, including *gag* and *pol*, respectively. The *gag* (group-specific antigen) encodes three viral open reading frames (ORFs): the viral matrix (MA), capsid (CA), and nucleoproteins (NC) (Figure 1B). The enzymatic machinery of the vector consists of reverse transcriptase (RT), protease (PR), and integrase (IN), which are pre-packaged into the viral particle (Figure 1C). Lentivirus is an enveloped vector; the most commonly employed envelope is vesicular stomatitis virus protein G (VSV-G), characterized by its very broad cellular tropism^18^ and shown in Figures 1B and 1C. The virus enters the host cell *via* complex transduction events and interactions between the viral envelope and the host membrane receptor, followed by fusion and intake. Next, the reverse transcription reaction (RTR) occurs (reviewed by Kantor et al.^17^). The process is governed by the RT enzyme, pre-packaged into viral particles in functional form. The enzyme mediates an RT process, which converts the viral genomic (vg) RNA molecule first into ss and then into double-stranded (ds), linear viral DNA (vDNA). The dsDNA form is then imported into the cell’s nucleus and serves as the integration template. Next, the integration process is governed by the IN protein.^19^^,^^20^ Following integration, the proviral DNA (pDNA) becomes integrated into the host’s genome and is hence passed along to the host cell’s generations. Notably, lentivirus is capable of sustaining its life cycle without an active IN enzyme.

Not surprisingly, the fact that IN is dispensable for infection has been exploited for the development of so-called IN-deficient LVs (IDLVs), which provide important advantages over traditional IN-competent LV (ICLV) counterparts.^21^^,^^22^ Indeed, despite substantial advances in the safety of lentiviruses, the utility of integrating systems per se is hampered by an elevated risk of insertional mutagenesis.^17^ IDLVs can be assembled by introducing nonpleiotropic mutations within the IN ORF.^23^ These mutations have been demonstrated to specifically disrupt only the integration reaction, without affecting other steps of the HIV-1 life cycle.^23^

We previously showed that IDLV vectors are capable of being expressed *ex vivo and in vivo*; nevertheless, the expression levels have been demonstrated to be significantly lower compared to ICLV counterparts.^21^^,^^22^^,^^24^ We showed that the low levels of IDLV-genome expression are attributed to the formation of a repressive, silencing chromatin organization around the episomal DNA (eDNA).^22^ Furthermore, we reported that the reduced levels of IDLV expression can be rescued by removing repressive elements from the viral cassette or in trans. On that note, we demonstrated that histone deacetylase inhibitors (HDACi) can potently activate transgene expression from IDLVs in both dividing and nondividing cells.^22^ Furthermore, we showed that the depletion of negative transcription elements (NTEs) located within the U3′-long terminal repeat region results in substantial upregulation of viral expression both *in vitro* and *in vivo*.^21^^,^^24^

Overall, IDLVs are an attractive means for transient delivery, including programmable nucleases into human cells. On that note, we recently developed an all-in-one IDLV vector with increased production and expression efficacy. We demonstrated that CRISPR-Cas9 delivered by this vector can mediate rapid and sustained gene editing in human cells and post-mitotic brain neurons *in vivo*.^24^^,^^25^ Furthermore, we reported that the expression of IDLV-delivered Cas9/guide RNA (gRNA) may lead to significantly lower levels of undesirable off-target perturbations compared to conventional ICLVs.^24^

### AAVs: Basic biology and engineering

**Figure 2:**
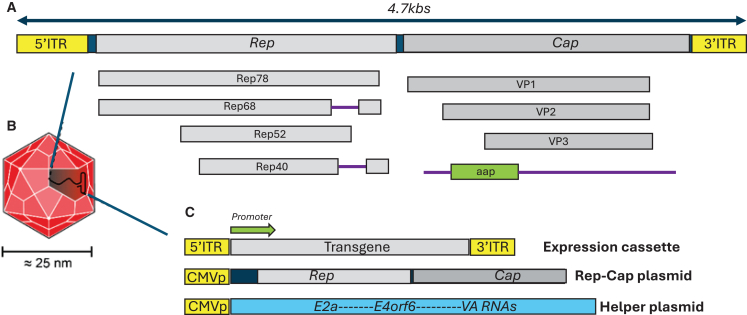
Overview of adeno-associated vectors (A) Simplified schematic of the wild-type AAV genome. (B) Simplified schematic of AAV particles. The size of the viral particles is indicated. Plasmids used to package AAV vectors are also illustrated. (C) AAV packaging system. The following plasmids are highlighted: the transgenic, helper (expressing E2a, E4orf6, and VA RNA), and *rep-cap* cassettes. The transgenic plasmid harbors inverted terminal repeat (ITR) sequences and the transgene of interest.

Adeno-associated viruses belong to the *Dependovirus* genus of the *Parvoviridae* family. As hinted by the genus name, the virus is defective, and its replication and life cycle are generally dependent on and governed by coinfection with a helper virus (either adenovirus or HSV-1) (reviewed by Li et al.^26^). The packaging-competent form of the viral genome is denoted by a 4.7 kb ssDNA. The wild-type AAV virus encodes a total of eight proteins, flanked by a pair of 146-bp inverted terminal repeats (ITRs). The *rep* and *cap* genes encode the replication-enabling enzymes and capsid-producing structural proteins, respectively.^26^ The *rep* gene generates four isoforms of the Rep protein, each combining two promoters and two splice isoforms (Figure 2A). The two slightly longer variants, namely Rep78 and Rep 68, are responsible for viral replication and integration, while the shorter Rep52 and Rep40 variants govern virion packaging. The *cap* gene encodes three capsid products: VP1, VP2, and VP3 (Figure 2A). A full discussion on the combination of transcriptional and translational pathways governing the expression of these products is beyond the scope of this review. However, in brief, VP1, VP2, and VP3 are translated at a ratio of 1:1:10, respectively.^27^ Sixty copies of VP1, VP2, and VP3 units, maintaining the same ratio, make up each icosahedral viral vector shell (Figure 2B). Assembly activating protein (AAP) is generated by a cryptic, unstructured genomic sequence located within the *cap* ORF (Figure 2A). This protein serves as a chaperone and is responsible for importing capsid proteins into the host cell’s nucleolus, where viral particle assembly occurs.^28^

AAV produces no envelope protein; therefore, the structural proteins composing the virion capsid determine its cell- and tissue-specificity, as well as tropism. Presently, over 150 AAV serotypes and capsids have been discovered and engineered, with AAV2.2 being the most utilized.^29^ The field of capsid engineering has greatly flourished in recent years, resulting in the generation of a remarkable variety of novel viral capsids (reviewed by Gao et al.^30^). The viral serotypes are usually engineered by altering the packaging cassette so that it contains the *cap* product from the variant of interest along with the *rep* product from serotype 2, while preserving AAV2 *rep*-binding sites in the viral ITRs within the transgene-harboring cassette. The resulting serotype is designated using a period; for example, AAV2.9 highlights a vector carrying the genome (*rep* gene) of serotype 2, packaged with the capsid from isoform 9. This particular serotype has shown improved transduction in neuronal cells that are not robustly infected by AAV2.2. Furthermore, it is spread more widely in the brain, accounting for more efficient transduction rates.^31^ The key studies utilizing AAV engineering methods, such as directed evolution, rational design, and in silico design, which have been developed to accelerate the discovery and translation of novel capsids, are systematically and comprehensively reviewed by Challis et al. and Nisanov et al.^32^^,^^33^

As mentioned earlier, over the last 30 years, remarkable efforts have been devoted to transforming AAVs into one of the platforms of choice for clinical gene therapy research. Several key milestones have contributed to progress toward this goal. First, it was demonstrated that the stem-loop-assembled repeats on both ends of the viral cassette (ITRs) are the only *cis-acting* structures required for both viral replication and packaging (reviewed by Rittiner et al.^34^). This understanding led to the establishment of a packaging system that supplied the *rep* and *cap* cDNAs in trans. The removal of the *rep-cap* sequences from the expression cassette allowed for an increase in the packaging capacity of the virus, bringing it close to 4.7 kb. Moreover, deletion of the *rep-cap* sequences from the viral plasmid was important to prevent reconstitution of the wild-type AAV genome during the production phase.^34^ Importantly, transferring the AAV *rep-cap* genes to the packaging plasmid enabled evasion of its inherent integration competence into human ch19.^35^ In fact, it was demonstrated that the wild-type AAV’s Rep78/Rep68 products are essential for viral targeted integration into ch19.^35^ Instead, the AAV genome appears to integrate randomly at a relatively low frequency (∼1%), with the vast majority of DNA being maintained as episomes.^36^^,^^37^ Second, the helper-associated function required for viral replication and production was initially supplied by infecting the producer cells with adenovirus or HSV-1. Nonetheless, this approach resulted in contamination of the viral preparations with the corresponding sequences. To circumvent this caveat, researchers engineered a separate plasmid carrying only the necessary helper genes: *E1a*, *E1b*, *E2a*, *E4orf6*, and viral-associated RNAs.^38^ Notably, HEK293T cells, which are most commonly used for prepping AAVs, already have *E1a* and *E1b* genes; therefore, these sequences are not included in the helper plasmid^38^ (Figure 2C). The optimized AAV preparation schedule thus uses three plasmids: the vector/expression plasmid carrying a transgene or transgenes of interest, the *rep-cap* plasmid, and the helper plasmid. These plasmids are usually introduced into the producer cells (HEK293/T) by the transient transfection method to produce viable AAV preparations^38^ (Figure 2C). This significant improvement over older production methods has enabled large-scale, clinical-grade manufacturing of the recombinant vector for a variety of gene therapy applications.

## CRISPR-Cas editing systems

**Figure 3:**
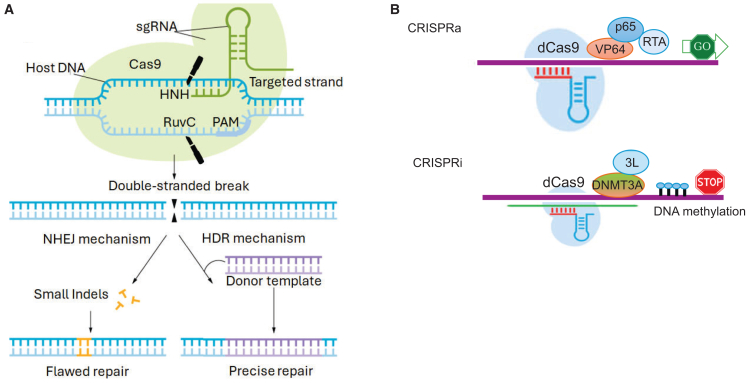
Applications of clustered regularly interspaced short palindromic repeats (CRISPR) technology (A) Double-stranded DNA breaks are introduced by active Cas9 and repaired *via* nonhomologous end-joining (NHEJ), creating insertions and deletions (Indels). Alternatively, if a dsDNA donor template is supplied, the dsDNA break can be resolved via homologous recombination, resulting in targeted insertion. (B) Strategies for epigenetic activation and repression of genes using CRISPR activation and CRISPR interference (CRISPRa and CRISPRi, respectively) fusion proteins. For gene activation, fusions containing the catalytic domains of transcriptional activators, including VP64, p65, and RTA, are used. An example of transcriptional repression is achieved by fusing dCas9 with DNA methyltransferases DNMT3A and DNMT3L. These de novo methyltransferases mediate DNA methylation at CpG sites, which in turn recruits inhibitory methyl-CpG-binding proteins.

The CRISPR-Cas system has recently emerged as a revolutionary tool for a broad range of gene therapy applications. Despite the immense complexity of CRISPR-Cas organization, all systems share CRISPR RNA, including gRNA and *trans*-activating RNA (tracrRNA), which define on-target specificity^39^ (Figure 3A). The most studied and utilized gene editing systems for applications in humans are engineered from the class II—types II, V, and VI CRISPR-Cas—which operate as a single protein. In contrast, class I Cas endonucleases act as multi-subunit complexes and are therefore less adaptable for applications in humans.^40^ For gene editing applications, the gRNA and tracrRNA are combined into one synthetic gRNA (sgRNA), which streamlines delivery (Figure 3A). A Cas enzyme performs a genome-wide search, during which protospacer-adjacent motif (PAM) recognition initiates local unwinding, supporting sgRNA binding to the targeted nucleic acid at the protospacer site. Next, the catalytic domain(s) of the Cas endonuclease cleaves the DNA or RNA strands of the targeted sequence, depending on the CRISPR-Cas system) (Figure 3A).

CRISPR-Cas has greatly transformed the gene editing repertoire, as the system can recognize its targets through simple Watson-Crick-Franklin complementation, which occurs between the protospacer and a sgRNA.^41^ This allows researchers to design CRISPR-Cas to align and cleave different DNA loci simply by altering the sequence of the sgRNA molecule (∼20 nucleotides), without necessitating the engineering of a new Cas enzyme (Figure 3A). In mammalian cells, dsDNA breaks generated as a result of Cas cleavage are repaired by nonhomologous end-joining (NHEJ) or homology-directed repair (HDR)^42^ and Figure 3A.

**Figure 4:**
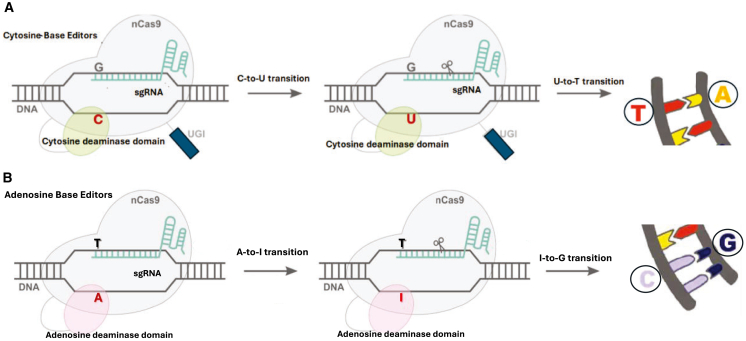
Base editing (A) The catalytically inactive dead (dCas9), paired with cytosine base editors, catalyzes the conversion of all cytosines within a 5–10 nucleotide window to uracils. During replication, uracil is read as thymine, completing the C:G to T:A conversion (highlighted in the upper-right image). (B) Adenosine base editors (ABEs) catalyze the conversion of all adenosines within a 5–10 nucleotide window to inosines. During replication, inosine is read as guanine, completing the A:T to G:C conversion (highlighted in the lower-right image).

One of the main limitations of employing active Cas endonucleases includes dsDNA break (DSB)-induced toxicity and the associated off-target cleavages,^43^^,^^44^ as well as variable and unpredictable editing results.^45^ These limitations have prompted the development and engineering of repurposed CRISPR-Cas technologies that improve specificity, versatility, and precision. The repurposed CRISPR-Cas editors combine the binding activity of catalytically impaired Cas enzymes with catalytic domains of transcription activators (CRISPR activation, or CRISPRa), repressors (CRISPR interference, or CRISPRi) (Figures 3B, 7 and 8), DNA deaminases (base editing [BE]) (Figure 4), RTs (prime editing) (Figure 6), and DNA INs (NHEJ-mediated insertions and deletions [indels]) to introduce new competencies for genomic perturbations. A detailed description of these technologies is summarized by Rittiner et al.^34^ Here, we present a brief overview of these technologies and their related clinical applications and discuss in more detail the translational aspect of conventional and repurposed CRISPR-Cas-based machineries for achieving permanent or transient manipulations of nucleic acids.

## Next-generation CRISPR-Cas technologies

### BE

BE technologies provide more efficient and precise methods for introducing single-base correction compared with conventional gene editing counterparts (reviewed by Rittiner et al. and Rittiner and Cumaran et al.^34^^,^^46^) (Figure 4). Unlike conventional CRISPR-Cas, which uses active Cas enzymes, BEs cleave only one DNA strand, as the nickase Cas (nCas9) is employed in most of these systems. As such, they create no DNA breaks; instead, the deaminase-active domain facilitates base pair conversions at specific genomic sites upon sgRNA hybridization. Cytosine base editors (CBEs) can efficiently convert a C:G to a T:A base pair,^47^ while adenosine BEs (ABEs) can alter an A:T to G:C pair^48^ (Figures 4A and 4B). Further development of BE technology has resulted in BEs capable of combining cytosine and adenosine deaminases in an all-in-one system.^49^^,^^50^^,^^51^ Most recently, the technology has evolved to facilitate efficient transversion corrections, e.g., C-to-G and A-to-C, complementing the editing scope of the transition-based systems mentioned above (reviewed by Villiger et al.^52^). Furthermore, Gupta and colleagues recently reported the development of DNA ADP-ribosylation-based editing tools capable of achieving a broader range of transversion mutations, including T>A, T>G, and T>C edits.^53^ Even with the active-window limitations characteristic of most BE systems, the current editors can amend up to 95% of all pathogenic mutations found within genes.

Notably, the residing time of Cas bound to DNA plays a crucial role in off-target preferences and rates. In fact, long-term or permanently expressed nick-competent Cas enzymes may greatly facilitate undesirable off-target and bystander effects of BEs, hindering their utility for clinical applications that require elevated levels of specificity and precision. More generally, the rise in promiscuous interactions with off-target genes due to excess gRNA/Cas delivered via integrating systems (e.g., LV) is well-documented.^24^ As such, transient (episomal) delivery platforms, such as IDLV and AAV, permanently modify the targeted loci using a “hit and run” strategy in dividing cells, which is beneficial for high-precision BE applications. To mitigate the undesirable genomic and transcriptomic off-target effects of BEs and other editing systems, several inducible CRISPR-Cas systems have been engineered.^54^^,^^55^^,^^56^ For example, Zeng and colleagues reported the development of a split ABE (sABE) design with inducible Cas-deaminase activity,^57^ achieved by integrating a chemically induced dimerization (CID) system.^58^ They demonstrated that Cas-ABE can be split into two parts: one fused to FK506-binding protein 3 (FKBP3) and the other to FKBP-rapamycin binding (FRB) protein. These two inactive ABE components can be reassembled into an active form upon rapamycin-induced FRB-FKBP3 heterodimerization.^57^ The inducible system, delivered via dual AAVs, was able to mediate efficient conversion of a single A•T base pair to a G•C base pair in the *Pcsk9* gene in mouse liver, demonstrating *in vivo* CID-controlled DNA BE.^57^ Similarly, Berríos and colleagues reported the development of a CBE-inducible system.^59^ It must be noted that the design of inducible systems generally comes with the cost of increasing the size of the genetic payload. In fact, all of these systems are too bulky to support delivery using a single AAV vector.

As mentioned above, the transgenic cargoes must be less than 4.7 kbs in order to be efficiently packaged into AAV virions, which limits the potential scope of AAVs as delivery vehicles for gene editing agents. To circumvent this limitation, several dual-vector approaches have been devised, including *trans*-splicing, fragmented, overlapping, and hybrid strategies (reviewed by McClements et al.^60^). Here are several examples of mRNA- and protein-mediated split systems. Riedmayr and colleagues recently developed a dual AAV-CRISPR-Cas vector based on reconstitution via mRNA *trans*-splicing (REVeRT).^61^ They demonstrated that the REVeRT system is flexible in its split site and can efficiently and robustly function in numerous *in vitro* models, in human organoids, and *in vivo*. Furthermore, REVeRT can also functionally reconstitute a CRISPRa system targeting genes in various tissues and organs in single or multiplexed settings.^61^

Another example of the mRNA *trans*-splicing method is recent work from Jin-Soo Kim’s laboratory. This group developed a BE approach to deliver the sABE and CBE modules, using a *trans*-splicing AAV vector, to muscle cells in a mouse model of Duchenne muscular dystrophy (DMD) to correct a nonsense mutation in the *Dmd* gene.^62^ Importantly, intramuscular administration of these vectors into a mouse model of DMD yielded about 4% editing,^62^ thus demonstrating the therapeutic potential of BE in adult animals.

Notably, the most widely used strategy for CRISPR-Cas delivery relies on protein-level split-intein systems (reviewed by Bengtsson et al. and Mittas et al.^63^^,^^64^). This approach utilizes a self-splicing protein mechanism in which two split-Cas segments are assembled, folding themselves into an active enzyme. The first application of this system in BE was for correcting murine phenylketonuria-associated phenotypes.^65^^,^^66^ To note, the split intein-protein approach, on average, resulted in 5-fold higher BE efficiency across multiple tissues in rodents when compared to RNA *trans*-splicing^66^ The dual AAV system combining the split-intein strategy has achieved BE efficiencies ranging from 10% to 50% across different organs, including the CNS, eye, liver, heart, and muscle.^67^^,^^68^^,^^69^

**Figure 5:**
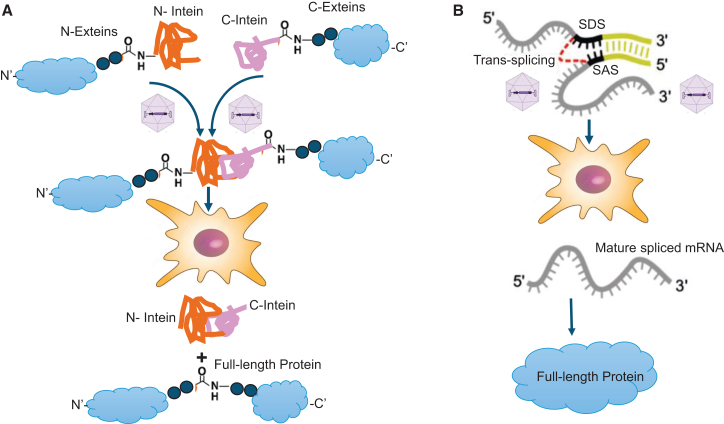
*Trans*-splicing mechanism reaction by split inteins (A) Protein *trans*-splicing. The N-intein is fused to the C-terminal end of the N-extein, while the complementary C-intein is located at the N-terminal end of the C-extein. Upon assembly of the two intein fragments, a splicing reaction occurs in which the intein excises itself from the precursor protein and simultaneously ligates the exteins via a peptide bond. (B) *Trans*-splicing at the mRNA level. SDS, splice donor site; SAS, splice acceptor site.

More recently, Koblan and colleagues applied the dual-AAV-BE in a mouse model of Hutchinson-Gilford progeria syndrome (HGPS) to correct the C:G-to-T:A mutation in the *LMNA* gene responsible for the disease.^70^ The authors reported up to 30% correction of the *LMNA* mutation in heart tissue, which resulted in a substantial reduction in progerin protein levels.^70^ Furthermore, Schwank’s group developed a split-intein-AAV2.9 tool to deliver an ABE system targeting the *Pcsk9* gene in rodents.^71^ They reported up to 50% BE in the liver, accompanied by an approximately 7-fold reduction in serum *PCSK9* protein levels and more than a 3-fold reduction in serum cholesterol.^71^ The dual-AAV system has also been applied in the rodent CNS to reduce mutant Huntington (HTT) expression, thereby rescuing pathological phenotypes associated with Niemann-Pick disease.^72^ Additionally, this approach was used to create a nonsense codon in the *SOD1* gene, which delayed disease progression in a mouse model of amyotrophic lateral sclerosis (ALS).^73^ The schematic description of mRNA- and protein-level split systems is illustrated in Figure 5.

### Prime Editing

**Figure 6:**
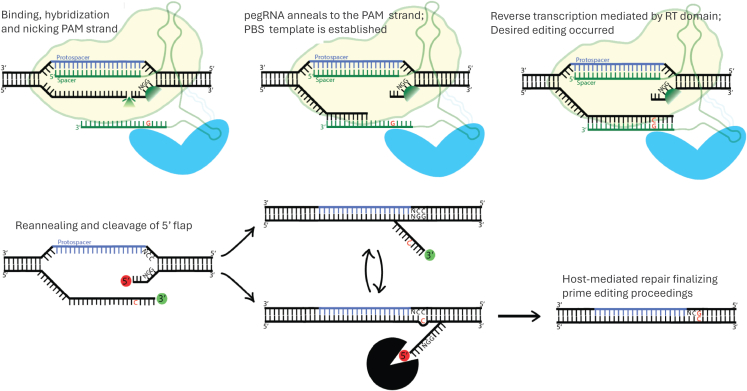
Prime editing Proposed mechanism of prime editing: the 5′ end of the pegRNA pairs with the protospacer sequence of the complementary DNA, while the nontargeted PAM strand is nicked. The nicked strand hybridizes with the primer-binding site (PBS) at the 3′ end of the pegRNA. The pegRNA serves as a template for RT reaction, which uses the newly created 3′-OH to initiate DNA extension. The targeted sequences then contain two redundant PAM strands, or “flaps.” The unedited 5′ flap is preferentially degraded, allowing the edited 3′ flap to anneal to the nonPAM (targeted) strand. Editing is completed by the desired insertion within the nonPAM strand.

To expand on the gene editing tools, David Liu’s laboratory developed a creative approach to gene editing, coined prime editing (PE)^74^ (reviewed by Zeng et al.^75^; Figure 6). The PE system is composed of a nickase, nCas9, fused with an engineered RT enzyme. Notably, the PE gRNA (pegRNA) differs substantially from conventional sgRNA. Furthermore, the pegRNA fulfills a dual role: it operates as a conventional gRNA for attracting the nCas9 protein and as a primer-binding site (PBS) template for the Cas9-attached RT domain (Figure 6). Briefly, the pegRNA is pre-complexed with the nCas9-RT protein, forming an RNP complex. This RNP then scans the genome for a compatible PAM sequence and engages in base pairing between the protospacer region of the pegRNA and the complementary DNA strand, after which DNA unwinding and nicking in the edited strand occur. The free 3′ end of the pegRNA then acts as a PBS, complementing the edited strand that has been nicked. The cleavage creates a free (3′-OH) end on the nicked strand, which can then be recognized and extended by the RT enzyme, using the PBS-attached region on the pegRNA as its template^74^ (Figure 6). The result of this process is two alternative DNA extensions, or “flaps”: the edited 3′ flap that was just created via RT-mediated replication, and the unedited, naive 5′ flap. The competing flapped DNA sequences are theoretically capable of complementing and hybridizing with the nontarget DNA sequence; however, the naive 5′ flap is thermodynamically favored to align over the created, edited flap. However, the 5′ flap is favorably degraded by cellular enzymes, which are quite abundant due to their role in lagging-strand DNA synthesis. As a result, the 5′ flap is quickly degraded, and the inserted 3′ DNA flap is then ligated (Figure 6). The consequence of the above steps is the formation of a heteroduplex with one DNA strand being edited and the other unedited. However, it is important to note that no direct experimental evidence or structural data (e.g., crystal or cryo-EM structures) have definitively confirmed this sequence of events in the context of PE. An additional nick in the nonedited (target) strand can be introduced by the same nickase if a separate gRNA is provided. That guide, dubbed the nicking sgRNA or ngRNA, will direct the PE complex to the nonedited strand. In the PE3 strategy, an additional nicking sgRNA is provided to encourage the cellular machinery to use the edited flap for repair.

The design of the ngRNA is specifically selected to target the sequence present only in the edited DNA strand, thereby preventing nicking of strands that have not undergone the desired edit. This design further reduces the probability of producing undesired indels by avoiding double-strand breaks at unedited loci. The improved PE system has been given a new name: PE3b.^74^ PE3b has been shown to efficiently mediate targeted indels of up to 150 bp, as well as support all types of point mutations, without necessitating DSBs. The efficiency of PE3b editors in cells *in vitro* ranges from ∼25% to 50%, with a 1%–3% rate of indels.^74^

In conclusion, the advantages of PE over BE are numerous: (1) no operational-window restriction; as such, no undesirable “bystander” editing; (2) significantly lesser PAM requirements (due to the customized/varied length of the selected RT template); and (3) pegRNA mediates ∼5-fold lower rates of undesirable off-target editing compared with its sgRNA counterpart.

The potential impact of PE technology on gene editing is enormous, underscored by its ability to theoretically reverse 90% of all known pathogenic mutations and disease-associated genetic variants (reviewed by Challis et al.^32^). Nevertheless, the size of most current PE systems is quite large; in fact, PEs are, on average, ∼1.5–2 kb bulkier than the corresponding BE systems. This creates a hurdle on the delivery end, as PE systems also need to be split into two vectors for successful gene transfer. Consistent with this notion, prime editors targeting *in vivo* applications have been engineered and utilized using split-intein AAV configuration.^76^^,^^77^^,^^78^ For example, Bock and colleagues reported 15% PE at the *Dnmt1* site in the mouse liver using two split-intein AAV2.8 vectors.^79^

It has to be noted, however, that the dual AAV systems designed to deliver PE tools currently yield significantly lower editing efficiency compared to their AAV counterparts using active Cas or BEs. However, recent advances in engineering of the RT moiety have targeted the development of more efficient and stable variants, including codon optimization and removal of the unnecessary RNase H domain in the RT enzyme, leading to size-minimized PEs that are more suited for AAV packaging.^30^^,^^80^ For example, a phage-assisted protein evolution approach led to shorter and more efficient constructs, dubbed PE6.^81^ In this study, Doman and colleagues discovered that different RTs may operate in different types of editing and exploited this insight to design a novel RT enzyme that was able to outperform the previous PEmax counterpart. In fact, the engineered and truncated PEmaxΔRNaseH variant was found to be more suitable for a split-intein-AAV delivery system.^82^^,^^83^ In addition, AAV-delivered PE6 enables longer insertions to be installed *in vivo*.^81^

### Epigenetic editing

In the past decade, the fusion of dead/inactive Cas (dCas) with various regulatory domains has provided researchers with unprecedented control over gene expression *in vitro* and *in vivo*. Here, we evaluate the current status of dCas9-based epigenetic systems.

### DNA methylation

The cytosine (C) base in the 5′ position of DNA can be methylated by DNA methyltransferases (DNMTs); in mammals, this modification occurs only when the C nucleotide is part of the specific two-base palindromic sequence CpG^84^ (Figure 3B). Notably, DNA methylation is a highly mutagenic process, as spontaneous deamination of 5-methylcytosine (5′-mC) produces thymine, thus converting the CpG pair to TpG. In fact, over evolutionary time, CpGs were effectively removed from the human genome by precisely this mechanism. The remaining sites, referred to as “DNA CpG islands (CGIs),” are enriched in the promoter regions of genes, where their methylation mediates stable and heritable transcriptional repression.^85^

The dysregulation of DNA methylation is the primary cause of multiple genetic diseases. For example, DNA methylation at CGI within the *SNCA* intron 1 region has been reported as a regulatory mechanism of *SNCA* gene transcription, and changes of methylation levels at this region are linked to Parkinson’s disease (PD), dementia with Lewy body (DLB), and other synucleinopathies.^86^^,^^87^^,^^88^ We and other laboratories have recently reported that *SNCA* intron 1-targeted DNA methylation can be used for fine-tuned downregulation of *SNCA* levels.^87^^,^^88^^,^^89^^,^^90^ Our epigenome-editing approach for PD is based on a novel and engineered LV carrying CRISPR-dCas9 fused with the catalytic domain of the de novo DNMT3A enzyme.^87^ Applying the LV-gRNA/dCas9-DNMT3A system to human induced pluripotent stem cell (hiPSC)-derived dopaminergic (DA) neurons from a patient with PD with *SNCA* triplication resulted in downregulation of *SNCA* RNA and protein governed by targeted methylation at the intron 1 region. Moreover, the reduction in *SNCA* levels rescued PD-related cellular phenotypic characteristics, e.g., mitochondrial reactive oxygen species (ROS) production and cellular viability^87^ (Figure 3B). Notably, the dCas9/DNMT3A activity can be substantially upregulated by additional fusion of the DNMT3A cofactor, DNMT3L^91^ (Figure 3B). In addition, DMTN domains other than DNMT3A have also been connected to the dCas9 carrier with similar outcomes, including the prokaryotic DNMT MQ1 enzyme.^92^

### Histone modifications

**Figure 7:**
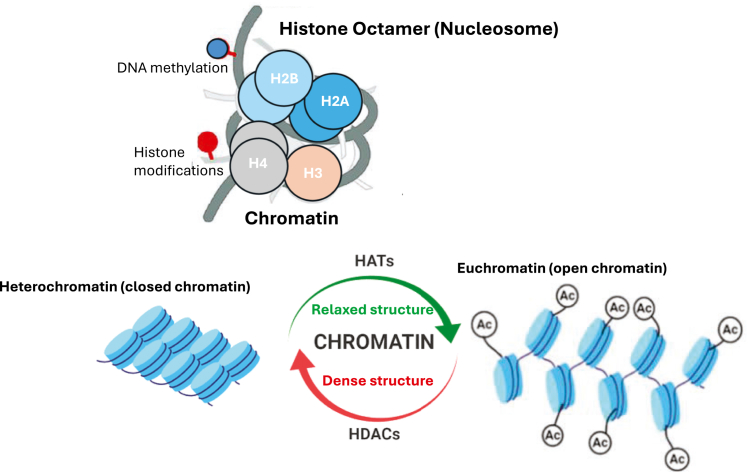
Chromatin structure and remodeling The structure of the octamer is illustrated. The octamer, the basic unit of the nucleosome, consists of two copies of each of the 4 core histone proteins: H2A, H2B, H3, and H4. Two states of genomic chromatin organization are shown: (1) heterochromatin, characterized by tightly packed and condensed DNA containing transcriptionally inactive sequences; and (*ii*) euchromatin, which consists of more loosely packed DNA and is associated with transcriptionally active regions. Several modifications that alter chromatin structure are highlighted. Histone acetylation and deacetylation regulate gene expression through the activities of histone acetyltransferases (HATs) and histone deacetylases (HDACs).

In nature, DNA molecules do not exist as “naked strings” but are wrapped around histones and histone-like proteins (Figure 7). Histone amino-terminal tails, which protrude from the globular histone-DNA core (called the nucleosome), are subjected to an extensive array of chemical modifications, including methylation, acetylation, phosphorylation, and others (reviewed by Razin et al. and Allis et al.^93^^,^^94^). These modifications form chromatin structure, which in turn regulates the accessibility of nucleosomes to binding proteins and subsequently contributes to the control of all key cellular processes, including transcription, replication, and the DNA-damage response.^95^^,^^96^

As an example, histone acetylation, which occurs at multiple K-residues across histones, neutralizes the lysine’s positive charge, thus lessening the association of the DNA-histone subunits (Figure 7). This event can lead to increased DNA accessibility and gene activation. In contrast to acetylation, histone methylation exerts profoundly diverse, often opposing effects on gene expression.^93^^,^^94^

**Figure 8:**
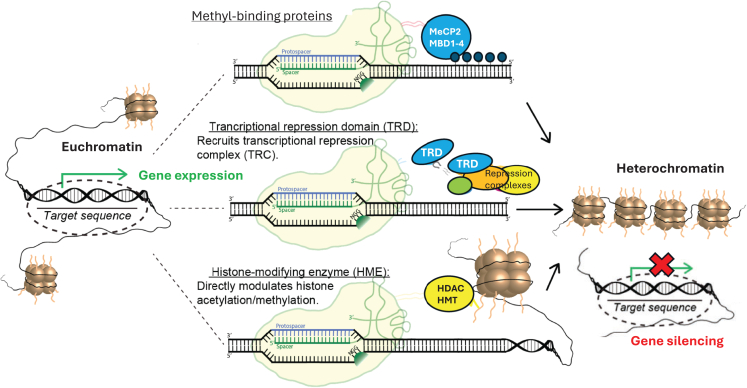
Epigenome editing The mechanisms illustrate approaches for epigenetic silencing. For example, dCas9 fused to methyl-CpG-binding domain (MBD) proteins or MeCP2 governs DNA methylation at CpG sites, which in turn leads to transcriptional repression. Alternatively, a transcriptional repression domain (TRD) can be fused to dCas9, resulting in the direct recruitment of transcriptional repressors. Finally, multiple forms of inhibitory histone-modifying enzymes can be fused to dCas9, altering histone acetylation and methylation, thereby establishing closed, transcriptionally inaccessible heterochromatin structures.

The repurposed CRISPR-Cas tools have been engineered for bidirectional manipulation of the above histone modifications. Notably, CRISPR-Cas-mediated transcriptional changes can be achieved using the dCas and sgRNA complex alone. In fact, many laboratories have demonstrated that the mere binding of dCas9 to promoters and other regulatory regions, including 5′-UTRs and introns, can efficiently downregulate transcription by sterically hindering the RNA polymerase II enzyme.^97^^,^^98^ The CRISPR-dCas-mediated repression effect has been coined “CRISPR interference” (CRISPRi) (Figures 3B, 7, and 8).

Notably, gene-silencing efficiency can be greatly improved if dCas9 is fused to a transcriptional repressor domain (TRD) (Figure 8). The most commonly used is the Krüppel-associated box (KRAB), a relatively small protein found in many human zinc-finger transcription factors.^99^ Several laboratories have reported that transcriptional inhibition produced when dCas9 is fused to KRAB is greatly superior to that achieved using dCas9 protein alone.^100^^,^^101^ Most recently, we reported the development of a miniature AAV-repressor system consisting of the KRAB-MeCP2 (TRD) domains^102^^,^^103^ (Figure 3B). The coding sequence of the all-in-one AAV-dSaCas9-KRAB-MeCP2 (TRD) systems is only 3.5 kb long, which is well suited for AAV packaging capacity. Using this system, we demonstrated efficient and permanent repression of the *ApoE* gene, which has been identified as the strongest genetic risk factor for late-onset Alzheimer’s disease (LOAD).^102^^,^^103^

Most recently, Jonathan Weissman’s laboratory described coupled histone tail for autoinhibition release of methyltransferase (CHARM), a compact, enzyme-free epigenetic editing system capable of repressing transcriptional events through programmable DNA methylation. Delivered via an AAV vector, the system efficiently ablated prion protein (PrP) expression across the mouse brain. As such, this novel silencing platform potentially represents a broadly applicable strategy to suppress pathogenic proteins, including those implicated in prion disease and other neurodegenerative disorders.^104^

Unsurprisingly, epigenetic activation can be achieved using CRISPR-derived tools (CRISPRa), most often by directly fusing dCas to an activation domain of transcription activators, such as VP64 or p300,^105^^,^^106^^,^^107^ or to tripartite constructs, such as VPR or VPH^105^ (Figure 3B). This approach allows precise control of gene expression, making it a valuable tool for studying gene functions and for developing potential therapeutic interventions for a variety of diseases and disorders. Further details on CRISPRa are left to other comprehensive reviews.^108^^,^^109^

## Engineering of compact Cas orthologs

As mentioned above, single-molecule AAV systems offer critical advantages for research and clinical use. Accordingly, substantial efforts have recently been made to identify smaller orthologs of Cas9 proteins and to generate small engineered Cas9 variants suitable for AAV delivery (reviewed by Kantor et al. and Rittiner et al.^3^^,^^46^). The Cas9 nuclease from *Staphylococcus aureus* (SaCas9) and *Campylobacter jejuni* Cas9 (CjCas9) are commonly used in the single-AAV approach, as they have transgene sizes of 3.2 kb and 2.8 kb, respectively, which are well suited for packaging into an all-in-one AAV vector. Recently, such delivery systems have been used in ongoing clinical trials involving subretinal administration of AAV-SaCas9/multiplexed sgRNAs to delete a disease-causing mutation in the *CEP290* gene in patients suffering from Leber’s congenital amaurosis type 10 (LCA10)^110^ (Table S1). In addition, the discovery of other compact Cas9 orthologs, such as Nme2Cas9 and SauriCas9, has increased the number of Cas9 variants that can be packaged into a single AAV particle. These compact Cas9 variants have also broadened the targeting scope of AAV editing agents by creating more flexible PAM motifs (for example, NNGRRT for SaCas9 vs. NNNRRT for SauriCas9).^111^

Similarly, Cas12, as the effector protein of CRISPR-Cas class 2 and type V, provides a convenient tool for gene and epigenome editing manipulations. Type V Cas effectors resemble type II in the overall simple organization of the CRISPR-Cas loci.^112^ For example, Cas12 and Cas9 endonucleases share homologous RuvC-like catalytic domains. However, the Cas12 enzyme is capable of processing the precursor crRNA by itself, which requires tracrRNA and/or RNase III nuclease.^113^ Consequently, Cas12 systems are more suitable for multiplexed genome-editing perturbations. Furthermore, using Cas12 can slightly reduce the system’s bulkiness, supporting more efficient packaging into a single AAV molecule. Furthermore, Cas12 seems to exhibit higher specificity than Cas9, allowing for more precise gene editing.^114^

Recent developments in hypercompact, programmable nucleases for DNA perturbations have inspired promising innovations on the delivery end as well. The currently characterized hypercompact Cas systems include Cas12j (CasΦ, 700–800 aa),^115^ Cas12l (Casπ, 747 aa),^116^ Casλ (∼700 aa),^117^ Cas12f (former Cas14) (400–700 aa),^118^ and Cas12n (400–700 aa),^119^ along with the CRISPR ancestral proteins TnpB (∼400 aa),^120^ IscB (∼400 aa),^121^ and their eukaryotic descendant Fanzor (400–700 aa)^122^ (Table S1). Although most of these ultracompact endonucleases exhibit modest editing activity in their native configuration, protein evolution and engineering hold the potential to improve their gene-editing performance.^123^ Importantly, the use of these compact Cas12 proteins can alleviate the packaging limitations of the AAV system. A comprehensive catalog of naturally occurring and genetically engineered Cas proteins can be reviewed by Kantor et al.^3^

## Clinical applications of CRISPR-Cas gene editing systems

### *Ex vivo* approaches

The high efficiency, robust activity, and straightforwardness of CRISPR-Cas-based editing tools have promoted their rapid integration into clinical research (Table S1). The first clinical study based on a CRISPR-Cas technology was reported by Stadtmauer et al.^124^ This first-in-human (FIH) phase 1 clinical trial was designed to evaluate the safety and feasibility of multiplexed CRISPR-Cas9 editing to engineer autologous T cells in three patients with refractory cancer. The autologous T cells were engineered by lentiviral transduction to delete two genes encoding the endogenous T cell receptor (TCR) chains: TCRα (*TRAC*) and TCRβ (*TRBC*). The removal of these genes was performed to reduce TCR mispairing and enhance the expression of a synthetic, cancer-specific TCR transgene (NY-ESO-1). Furthermore, a third gene, encoding programmed cell death protein 1 (PD-1; *PDCD1*), was removed by the researchers to improve the antitumor immune response. This study reported that *ex vivo* modified T cells were able to persist for 9 months following transfusion, suggesting the feasibility of the gene-editing approach for cancer immunotherapy.^124^ Similarly, early trials for *ex vivo* CRISPR-Cas9 editing and allogeneic transplantation of hematopoietic SCs (HSCs) were designed to delete CCR5, the chemokine receptor that permits infection with the HIV-1 virus (https://clinicaltrials.gov/study/NCT03164135) (Table S1). This clinical study reported stable engraftment of the CCR5−/− edited cells using LV, albeit with low levels of editing, which are deemed insufficient to achieve a cure for the disease.^125^ Building on these precedents, many other clinical trials have been designed to apply CRISPR-Cas tools in the area of cancer immunotherapy. Several of these trials used active^126^^,^^127^ or dCas9 endonucleases^128^ in *ex vivo* attempts to delete immunosuppressive genes. In one study, Chiesa and coworkers used a BE approach paired with LV delivery to generate universal, off-the-shelf CAR-T cells. The BE tools were applied in this study to inactivate three genes encoding the CD52 and CD7 receptors and the β chain of the αβ TCR to evade lymphodepleting serotherapy, CAR7 T cell fratricide, and graft-versus-host disease, respectively. The FIH phase 1 study then evaluated the safety of the edited cells in three children suffering from relapsed leukemia. The positive results of this study prompt further investigation of base-edited T cells for patients with relapsed leukemia^128^ (Table S1). In these clinical studies, researchers used LVs to engineer and deliver CRISPR-Cas tools. As mentioned above, *ex vivo* gene therapy applications heavily rely on LV delivery (see Figure 2). In fact, lentivirus may be a better fit in this context, as it can circumvent several limitations of simple retroviral vectors, including a significantly lower frequency of insertional mutagenesis, lower risk of oncogenicity and related toxicities, and higher virion stability and titers.^129^ Consistently, LV has recently been used to deliver active Cas9 enzyme to induce the permanent deletion of an antibody–drug conjugate in HSCs, which was performed to reduce toxic effects from treatment after HSC transplantation.^130^ Another highly attractive area for *ex vivo* CRISPR-Cas-gene therapy is the treatment of genetic blood diseases, including SCD and TDT, both of which involve mutations in the gene producing hemoglobin-β (*HBB*). In this context, CRISPR-Cas9-mediated DSB-induced deletions were used to disrupt *cis-acting* elements, such as the γ-globin (HbG) repressing factor, BCL11A, supporting the compensatory expression of fetal hemoglobin F (HBF).^131^ In 2023, the FDA and the UK’s Medicines and Healthcare Products Regulatory Agency (MHRA) approved exagamglogene autotemcel (brand name, Casgevy) as the first-in-class *ex vivo* CRISPR-Cas9 gene therapy to treat SCD and TDT (Table S1). Similarly, in 2023, the FDA granted approval to lovotibeglogene autotemcel, sold under the brand name Lyfgenia or LentiGlobin by Bluebird Bio, for the treatment of patients 12 years of age and older with SCD and a history of vaso-occlusive events.^132^ LentiGlobin comprises autologous transplantation of hematopoietic stem and progenitor cells transduced with a lentivirus encoding a modified β-globin gene (β^A-T87Q^) to produce antisickling hemoglobin (HbA^T87Q^).^132^ To note, this treatment is limited to gene augmentation therapy and not gene editing. In addition, an erythroid-specific knockdown of BCL11A delivered by an LV-encoded, microRNA-adapted short hairpin RNA has been demonstrated to reactivate the γ-globin gene and is currently in early clinical development.^133^ Most recently, the FDA has permitted a phase 1/2 trial investigating EDIT-301, Editas Medicine’s gene editing cell therapy for severe SCD and TDT (https://clinicaltrials.gov/study/NCT04853576). The safety and efficacy of a single dose of EDIT-301 in patients will be assessed in a planned open-label study called RUBY. EDIT-301 is an experimental autologous cell therapy in which CD34+ cells are genetically modified to promote γ-globin expression. γ-globin induction in EDIT-301 is attained through AsCas12a RNP-mediated editing of the distal CCAAT box of the HBG1 and HBG2 promoters^100^^,^^134^ (Table S1). Another phase 1/2 clinical trial, entitled “Transplantation of Clustered Regularly Interspaced Short Palindromic Repeats Modified Hematopoietic Progenitor Stem Cells (CRISPR_SCD001) in Patients with Severe Sickle Cell Disease,” initiated by Mark Walters of the University of California, San Francisco, uses an RNP-Cas9-induced DSB mechanism to stimulate homology-directed recombination to correct the SCD mutations in HBB (https://clinicaltrials.gov/study/NCT04774536) (Table S1). Most recently, CRISPR Therapeutics AG initiated an open-label, FIH study in patients with type 1 diabetes (T1D) to evaluate the safety, tolerability, and efficacy of the VCTX211 combination product. This trial (status: actively recruiting patients) applies Cas9-modified pancreatic endoderm cells derived from allogeneic PSCs for the treatment of diabetes mellitus (https://clinicaltrials.gov/study/NCT05565248). There are a few other examples of using LVs for *in vivo* gene editing outside of hematological diseases. Suh and colleagues used LV to subretinally administer ABE and sgRNA components to correct a premature nonsense codon in the *Rpe65* gene in a mouse model of Leber congenital amaurosis (LCA).^135^ A single dose of LV-ABE administered into the eye resulted in ∼15% BE correction and almost completely rescued visual function. The efficient delivery of LVs for *in vivo* administration into other organs, including the bone marrow, brain, and liver, has also been demonstrated, although these applications are limited to gene replacement therapy.^136^^,^^137^^,^^138^

It should be noted that preclinical studies and early clinical trials had suggested very low safety concerns associated with *ex vivo* gene therapy.^139^ Nevertheless, the potential risks of oncogenicity and genotoxicity, as well as the causes of direct or secondary malignancies, are now being comprehensively revisited as larger numbers of patients are treated and corresponding data are accrued. Notably, and unfortunately, 6 of 67 patients treated to slow the progression of cerebral adrenoleukodystrophy (CALD)—an X-linked rare inherited neurodegenerative disease affecting a peroxisomal fatty acid transporter central to fatty acid metabolism—who received Bluebird Bio’s FDA-approved LV-based gene therapy for CALD (elivaldogene autotemcel) developed myelodysplastic cancers.^140^ Skysona utilizes a self-inactivating and replication-defective LV, dubbed Lenti-D, that harbors the strong synthetic promoter MNDU3. The study reported that patients who developed blood cancers showed clonal expansion of cells containing the virus integrated into the *MECOM* and *PRDM16* proto-oncogenes. The problems may not have been solely due to the LV, the study reports.^141^^,^^142^ Similarly, Beam Therapeutics recently reported the death of a patient in a phase 1/2 trial assessing the BE therapy candidate BEAM-101.^143^ Nevertheless, the company stated that the death was likely caused by the preconditioning regimen preceding dosing and not the treatment. Beam-101 (BEACON) is an open-label, single-arm study evaluating the safety and efficacy of the administration of autologous base-edited CD34+ HSPCs in patients with severe SCD (Table S1) (https://clinicaltrials.gov/study/NCT05456880).

Notably, the edited cells were *ex vivo* treated using an RNP-electroporated-based protocol. In fact, nonviral methods such as electroporation have gained traction for *ex vivo* delivery into HSCs and T cells.^144^ Electroporation allows direct and efficient delivery of Cas protein and sgRNA in the form of an RNP complex. Importantly, as mentioned in this review, two large companies operating in the CRISPR-Cas9 space, Vertex and CRISPR Therapeutics, recently collaborated on the development of an *ex vivo* Cas9-enabled therapy, known as Casgevy (exagamglogene autotemcel), employing pre-packaged RNP complexes that specifically target the erythroid-specific enhancer of *BCL11A*, *ex vivo* delivered to HSCs by electroporation.^145^ Electroporation of CRISPR-Cas9 RNP, paired with a DNA-based donor template to facilitate HDR, has enabled precise and efficient nonviral genome editing of T cells.^146^ Notably, the electroporation method used in this study outperformed conventionally generated CAR T cells in a mouse model of ALL.^146^ Furthermore, this strategy allows enhanced control over the duration of component presence in cells while minimizing off-target effects and toxicity.^146^

### *In vivo* approaches

Thus far, *in vivo* CRISPR-based therapeutic approaches have centered around genetic diseases and disorders that affect tissues and organs that are relatively accessible for transduction, such as the liver, eye, and blood. It is worth noting that these “easy-to-target” tissues can also be readily and efficiently targeted *via* nonviral delivery methods.^14^^,^^147^ For example, a recent phase 1/2 clinical trial^145^ evaluated the safety and pharmacodynamic effects of single escalating doses of NTLA-2001 in patients with hereditary transthyretin amyloidosis (ATTR) with polyneuropathy (https://clinicaltrials.gov/study/NCT04601051) (Table S1). The interim results of this study reported that a single dose of LNPs encapsulating mRNA for Cas9 and sgRNA targeted transthyretin in the liver could safely and durably lower the load of toxic, misfolded protein by 80%–95%.^148^ The FDA recently cleared a phase 3 trial of this approach, making it the first *in vivo* CRISPR-Cas study to advance this far in treating ATTR disease. Similarly, a clinical trial using LNPs to deliver Cas9 nuclease and sgRNA to knock out kallikrein in patients with hereditary angioedema (HAE) also generated very promising interim results (Table S1). The data showed that following treatment, patients with HAE experienced a significant decrease in plasma kallikrein and related angioedema attacks (clinical trial: “NTLA-2002 in adults with hereditary angioedema (HAE) (NTLA-2002)” conducted by Intellia) (https://clinicaltrials.gov/study/NCT05120830). Another clinical study employing LNP particles has been designed to deliver BE agents to knock out the gene encoding proprotein convertase subtilisin/kexin type 9 (PCSK9) in patients with heterozygous familial hypercholesterolaemia (HeFH)^71^ (Table S1). This study aims to reduce low-density lipoprotein (LDL) levels, thereby lowering the risk of atherosclerotic cardiovascular disease (ASCVD). The first phase of this study is designed to determine the safety and pharmacodynamic profile of VERVE-101 (Verve Therapeutics). The study targets adult patients diagnosed with HeFH and established ASCVD (https://clinicaltrials.gov/study/NCT05398029).

The CRISPR-Cas has shown great promise in treating a variety of inherited or multifactorial ocular diseases. One study, mentioned above, utilized an all-in-one AAV vector to deliver both SaCas9 nuclease and sgRNAs in an attempt to delete the mutated *CEP290* gene in patients suffering from LCA10.^149^ The purpose of this study, initiated by Editas Medicine, was to evaluate the safety, tolerability, and efficacy of single escalating doses of EDIT-101 administered via subretinal AAV injection in participants with LCA10 caused by a homozygous or compound heterozygous mutation involving c.2991 + 1655A>G in intron 26 of the *CEP290* gene (LCA10-IVS26) (Table S1) (https://clinicaltrials.gov/study/NCT03872479. In this clinical trial, EDIT-101 (AAV2.5) was injected in 12 adults.^149^ The highly positive outcomes of this clinical trial, highlighted by high safety and improvements in photoreceptor function, support further research on *in vivo* CRISPR-Cas9 gene editing to treat inherited retinal degenerations due to the IVS26 variant of *CEP290* and other genetic causes. Another clinical study using AAV-mediated delivery was designed to target HIV-1 infection in patients on stable antiretroviral therapy (ART). The plan in this study was to assess the efficiency of gene editing for HIV-1-pDNA excision in the patients’ blood (https://clinicaltrials.gov/study/NCT05144386) (Table S1). In a different approach, CRISPRa-based gene therapy using dCas9-VPR-mediated transcriptional activation was recently carried out to treat a patient with DMD (Table S1). In this clinical trial,^150^ a 27-year-old patient with DMD was treated with AAV2.9-containing d*Sa*Cas9 fused to the VP64 activator; the transgene was engineered to upregulate cortical dystrophin levels. A high dose of AAV (1 × 10^14^ vg per kg of body weight) was used in this study. Tragically, the treated patient developed cardiac dysfunction and pericardial effusion, followed by acute respiratory distress syndrome (ARDS) and cardiac arrest 6 days after transgene treatment, and died 2 days later.^150^ Notably, despite severe diffuse alveolar damage, the therapeutic transgene’s expression in the liver was minimal, and there was no evidence of anti-AAV2.9 antibodies or effector T cell activity in the organs. The findings of this clinical trial (funded by Cure Rare Disease) may indicate that an innate immune reaction caused ARDS in a patient with advanced DMD treated with high-dose AAV gene therapy. In September 2025, Capsida Biotherapeutics informed the STXBP1 community that it had paused its phase 1/2 study after the first patient died. The trial, launched in July 2025, aimed to assess CAP-002 gene therapy in children with syntaxin-binding protein 1 (STXBP1) encephalopathy, a condition characterized by abnormal brain function and recurrent seizures (https://clinicaltrials.gov/study/NCT06983158) (Table S1). The company used its proprietary AAV capsid, designed to de-target the liver and dorsal root ganglia while crossing the blood-brain barrier (BBB) during intravenous (i.v.) administration of its antisense oligonucleotide therapy. Moreover, around the same time, the FDA announced that it was investigating the death of a patient who received Sarepta Therapeutics’ DMD gene therapy, Elevidys, which uses AAVrh74-micro-dystrophin. Elevidys was approved by the FDA in 2023 as the first gene therapy for pediatric patients aged 4 to 5 years with DMD.^151^ Nevertheless, Sarepta, in its own communique, elaborated on the situation and stated that the death of the patient was unrelated to Elevidys itself. However, earlier, in March 2025, the FDA revealed that it was looking into the death of another patient who received Elevidys.^151^ Unlike this case, another patient died following receipt of an investigational Sarepta gene therapy drug, dubbed SRP-9004, in a clinical trial for a different form of muscular dystrophy (https://clinicaltrials.gov/study/NCT06747273). Crucially, however, both SRP-9004 and Elevidys used the same AAV vector platform. All three patients whose deaths were linked to Sarepta’s gene therapy died from acute liver failure. Liver toxicity is a known side effect of AAV-based gene therapies.

Furthermore, an advantage of nonviral delivery methods, such as LNP therapies, is the potential for re-administration, which is contraindicated with AAV-delivered therapies given the immunogenicity of the vector.

Most recently, in a historic medical breakthrough, a child diagnosed with a rare metabolic disease known as severe carbamoyl phosphate synthetase 1 (CPS1) deficiency was successfully treated with a customized LNP-based ABE-CRISPR-Cas gene editing therapy by a team at the Children’s Hospital of Philadelphia (CHOP) and Penn Medicine. The infant, KJ, received two infusions at approximately 7 and 8 months of age.^152^ Remarkably, after the infusions, the patient was able to tolerate an increased amount of dietary protein and a reduced dose of a nitrogen-scavenger medication—half the starting dose—without unacceptable adverse events. KJ has also been able to recover from typical childhood illnesses, such as rhinovirus, without ammonia building up in his body.^152^ Longer follow-up is needed to fully evaluate the benefits of the therapy. It must be stressed that gene therapy approaches for diseases like CPS1 deficiency are truly transformative, as patients like KJ are typically treated with a liver transplant. However, to qualify for liver transplant surgery, patients need to be old enough and medically stable. During that time, episodes of increased ammonia can put them at risk for ongoing, lifelong neurologic damage or even death.

While additional research will be needed to address the safety concerns of AAV-based therapeutics, this vector platform remains a front-runner for *in vivo* gene therapy applications, including those targeting the CNS (reviewed by Kantor et al.^3^). On this note, in a major breakthrough for neurodegenerative diseases, uniQure N.V. announced positive topline data from its pivotal Phase 1/2 study of AMT-130, an investigational AAV2.5 gene therapy for HTT’s disease (https://clinicaltrials.gov/study/NCT05243017). The company reported that the high-dose AAV treatment significantly slowed disease progression, offering a potential disease-modifying solution for this devastating, inherited disorder. A comprehensive list of FDA-approved clinical studies involving CRISPR-Cas9-viral vector systems can be found in Table S1.

## AAV and LV delivery challenges and solutions

As mentioned earlier, a major limitation in the application of AAV vectors is their relatively small transgene capacity (up to approximately 4.7 kb), which makes it challenging (or outright impossible) to package bulky transgenes. Indeed, oversized constructs such as CRISPR-Cas, for the most part, require the use of two complementing AAVs, especially if *in vivo* transduction is desired. While the dual-vector strategy highlighted above has mediated therapeutic gene editing in mouse models of human disease, the low efficiency of vector reconstitution remains a major drawback of such approaches. Furthermore, split-vector methods are not very flexible and yield double the amount of immunogenic or pathogenic particles, thus raising serious safety concerns for translational applications.

From those perspectives, an all-in-one AAV delivery system would offer key advantages for research and clinical use by enhancing therapeutic effects, reducing virus-associated immunogenicity and related toxicities, and simplifying the manufacturing process. An all-in-one vector strategy can also lower the total dose of AAV required to achieve a therapeutic level of gene editing. Moreover, single-vector approaches might enable higher editing efficiencies in cells and tissues that are currently difficult to transduce by avoiding the need for simultaneous administration of multiple vectors. These advantages served as the impetus for the development of a single-AAV system capable of efficiently transferring a fully functional CRISPRi transgene to a cell and tissue of interest.^102^^,^^103^ A similar approach has been taken by Mahata and colleagues, who engineered a compact CRISPR-Cas-human mechanosensitive transactivation module to enable potent and versatile synthetic transcriptional control.^107^

Immune response against the capsids is another key bottleneck of AAV. This limitation arises from the fact that AAV is a nonenveloped virus, and its capsid is covered with amino acid residues (see above). Such anticapsid antibodies can neutralize viral particles even at relatively low concentrations, thereby blocking entry into target cells.^153^^,^^154^ This limitation means that AAV-mediated delivery is essentially a single-shot therapy and cannot be continually applied unless a nonself-reactive serotype is employed. On the same note, high levels of pre-existing neutralizing antibodies (50%–80%) against viral capsids have been detected in many human populations.^155^^,^^156^

Obviously, the issue of immunogenicity and related toxicities can be solved by excluding patients who are positive for neutralizing antibodies from clinical trials, but this approach is not an ideal solution. A more feasible strategy is engineering AAV capsids capable of escaping humoral immunity. For example, a novel capsid, CAM130, demonstrated diminished neutralizing activity against AAV2.1 while preserving comparable physical and functional titers and tissue tropism.^157^ Furthermore, some wild-type AAV serotypes have shown inherently lower prevalence of neutralizing antibodies in humans^158^ and therefore can be exploited as templates for capsid development. One example is the rational-based engineering of AAVrh.10, one of the least seropositive viral capsids.^159^ The novel AAVrh.10-S671A variant achieved superb transduction efficiency; importantly, it was significantly more resistant to neutralizing antibodies when tested in rodents.^160^ The shortcoming of this rational design-based engineering is its reliance on detailed understanding of capsid structures and antibody-binding epitopes. Furthermore, cross-reactivity of neutralizing antibodies against capsid residues, even if partial, may limit the success of this approach.

As mentioned earlier, the AAV vector is a transient system and typically does not integrate into the host genome. Notwithstanding, AAV carrying the CRISPR-Cas9 transgene has shown a significant increase in the rates of illegitimate integration.^161^^,^^162^ The possibility of AAV-CRISPR-Cas integration may lead to undesirable genotoxicity related to the disruption of an essential gene (i.e., silencing of a tumor suppressor gene or activation of an oncogene). Therefore, lowering the therapeutic dose must be the ultimate goal in this context as well. Achieving this may require a combination of vector engineering, CRISPR-Cas9 engineering, and related pharmacological strategies aimed at increasing vector transduction and expression efficiency, thereby mitigating the aforementioned side effects.

As such, future optimizations and improvements in AAV and other delivery systems will be required to advance CRISPR-Cas and other gene therapies. While further research may address the lingering safety concerns of AAV-based therapies highlighted above, new manufacturing processes will be needed to scale up production of these viral vectors to meet the growing demand. Large-scale production of homogeneous, high-purity AAV preparations at therapeutic doses (10^11^–10^13^ vg/kg) remains challenging. The presence of ITRs complicates plasmid replication and packaging, often resulting in heterogeneous vector populations (e.g., partial genomes and empty capsids) that may affect both efficacy and safety. In fact, multiple challenges in AAV production contribute to irregularities in titers, quality, and purity between different batches, and the overall process remains highly labor-intensive. Recent research has therefore focused on modifying the standard production workflow at the plasmid level to improve both titers and vector quality while reducing cost. Notably, a recent study showed that low-level transfection (using 1%–10% of the expression cassette) may substantially reduce the amount of transgene plasmid required compared with the standard transfection protocol, while simultaneously increasing *in vivo* transduction efficiency.^163^ Importantly, this strategy supports the manufacturing of yield-inhibiting cargoes and reduces plasmid contamination in preparations and in the tissues of mice receiving this vector.

Future manufacturing methods will need to aim for maximum quantity, quality, and scalability, while reducing or even eliminating the need for plasmid transfection. In that regard, numerous efforts have been directed toward establishing stable and inducible producer cell lines with these key characteristics (reviewed by Bulcha et al.^158^).

Still, despite the remarkable progress highlighted above, viral manufacturing remains one of the biggest economic challenges, limiting the use of AAV, LV, and other vectors as first-line treatments. Notably, the majority of early viral-based therapeutics were designed and manufactured within the context of rare diseases, either through local administration to specific tissues or *ex vivo* manipulation of cells. In those settings, small quantities of vector were sufficient, particularly as most gene therapy programs were still in the investigational new drug (IND)-enabling or early clinical stages of development. Now, with the shift beyond rare-disease applications, viral vector manufacturing requires rapid expansion to adequately address these clinical indications at the commercial level. As mentioned above, scalability of upstream processes is crucial for low-cost commercial manufacturing of AAVs, LVs, and other viral vector platforms, especially as they begin to expand into the nonrare disease space.

Despite the aforementioned progress, at present, infrastructure and logistics in the gene therapy industry are still heavily skewed toward transient-based transfection protocols. In fact, all approved AAV- and LV-based therapies are currently produced in adherent or suspension transient-transfection systems (reviewed by Bulcha et al.^158^). However, transient transfections demand infrastructure- and labor-costly settings and become increasingly ineffective at larger volumes. The baculovirus expression vector (BEV) is currently the most scalable and cost-effective system, as it can be scaled up to 2K L bioreactors.^164^ However, the insect cell–based BEV platform generates AAV particles with lower infectivity due to the lack of mammalian post-translational modification machinery, leading to higher doses being needed for therapeutic transduction.^165^^,^^166^

As highlighted above, unlike their AAV counterparts, LVs are the platform of choice for viral systems for *ex vivo* gene correction. However, LVs share similar challenges to AAVs in that the journey to advance them to the forefront of clinical gene therapy was not straightforward or deliberate. LVs are enveloped viral systems, which means they are fully competent upon budding from the cell. From a manufacturing standpoint, this represents a significant challenge, as the extracellular biomaterial needs to maintain this highly complex structure.

As mentioned above, LVs are pseudotyped with the G glycoprotein from vesicular stomatitis virus (VSV-G) and baculovirus gp64, with both envelopes being capable of conferring physical stability and high transducibility to viral particles and showing wide tissue and species tropism.^167^^,^^168^ However, a drawback of these pseudotypes is their sensitivity to inactivation by human complement.^169^ The complement-dependent inactivation of both VSV-G- and gp64-pseudotyped LVs could be circumvented by expressing complement regulatory proteins (CRPs) in the producer cells. In fact, Schauber-Plewa and colleagues demonstrated that the overexpression of native DAF/CD55 CRPs in the viral producer cell is an easy method to protect viral particles against complement inactivation.^170^ In an alternative approach, more recently, Hu and coworkers evaluated two serologically distinct novel viral envelopes derived from Chandipura (CNV-G) and Piry (PRV-G) vesiculoviruses.^171^ Both supported the generation of high-titer LVs with the capacity for high-efficiency gene transfer into various cell types from different species. Remarkably, both novel envelopes were found to be more resistant to inactivation by human serum complement compared to VSV-G.^171^

As mentioned above, VSV-G pseudotyped LVs signify a major advancement in the gene and immunotherapy field, fulfilling the promise of *ex vivo* gene therapies in hematological diseases such as β-thalassemia (Bluebird’s Zynteglo and Lyfgenia), CAR T cell therapies (Novartis’s Kymriah, CALD (Bluebird’s Skysona), and metastatic synovial sarcoma (Adaptimmune’s LLC Tecelra). Notwithstanding these historic breakthroughs, LVs supplemented with the VSV-G envelope do not provide efficient transduction of quiescent T cells, B cells, and HSCs, which hampers their application in gene and immunotherapy areas. Recently, Finkelstein and colleagues demonstrated that the LDL receptor (LDL-R) serves as the major entry port for VSV-G-pseudotyped LVs in human cells.^172^ The widespread expression of LDL-R accounts for the pantropism of VSV-G.^173^ However, LDL-R levels on unstimulated T, B, NK cells, as well as CD34^+^ cells are extremely low, which coincides with LV-VSV-G–mediated poor transduction in these four cell lineages. Importantly, triggering T cells via the TCR, or stimulating human CD34^+^ cells with early-acting cytokines, e.g., interleukin (IL)-3 and IL-6, remarkably upregulated LDL-R expression and allowed efficient LV-VSV-G-mediated transduction.^174^ In contrast, this study reports that B cell receptor stimulation augmented LDL-R expression only marginally, in agreement with poor LV-VSV-G transduction levels, suggesting that other LV-heterologous pseudotypes (e.g., measles virus envelope proteins [MV-LVs]) could be more efficient for gene transfer in these important resting gene therapy targets.^174^ In summary, manufacturing of viral vector-based therapies will likely remain a crucial point of differentiation in the coming years. Addressing the bottlenecks and challenges of viral vector manufacturing will be crucial for gaining regulatory approvals and achieving patient access to lifesaving gene therapy programs. Safety of viral vectors, including AAVs, remains a major concern for therapy development. While rAAV is favored among currently available viral vectors because of its safety profile, it is not without risks, especially at high doses or in specific patient populations. For example, widespread and efficient targeting of muscle tissue for DMD is likely to be achieved only with the use of systemic administration. Altering and improving AAV capsids to enhance tropism and reduce therapeutic doses will be essential for improved safety and efficacy using this route of administration (ROA). Although limb perfusion (LP) could serve as an alternative, it is unlikely to reach the therapeutic levels required to rescue disease pathology.^175^ However, combinations of ROAs might help capitalize on the advantages while concurrently minimizing the disadvantages. Another example is the use of AAV-delivered gene editing and other therapeutics for CNS disorders. Although new ROAs to confine AAV to the CNS space are being established and tested, the widespread and efficient transduction of the CNS seems to benefit from the finely arborized blood vessel supply of the brain, which is leveraged by IV AAV administration.^176^^,^^177^^,^^178^ In conclusion, progress in gene therapy has uncovered exciting new therapeutic avenues for many previously incurable diseases. However, strong and consistent efforts will be needed to overcome the challenges associated with gene therapy approaches to achieve their full therapeutic potential.

## Acknowledgments

We would like to thank Dr. Leanne Duke for technical assistance and for editing this manuscript. We also thank the editors and reviewers for their insightful comments, which greatly improved the manuscript. This work was funded by the Startup fund 204113-550-047352 provided by the Institute of Pediatric Diseases to B.K.

## Author contributions

B.K. wrote the manuscript. B.K., L.D., and P.G.B. discussed, edited, and prepared the manuscript.

## Declaration of interests

B.K. is a co-founder of CLAIRIgene LLC and Montserrat Inc.

## Supplemental information

Table S1. Ongoing clinical trials using CRISPR/Cas systems
